# Thank you to *The Lancet Regional Health - Western Pacific*’s clinical and statistical peer reviewers in 2023

**DOI:** 10.1016/j.lanwpc.2024.101038

**Published:** 2024-02-29

Emma Aarnio

Maya Abdelwahab

Adugna Abera

Lucas Guimarães Abreu

Laith Abu-Raddad

Thomas M Achenbach

Tim Adair

Nafees Ahmad

Bilal Ahmed

Murat Akova

Mohammed A Al Mahish

Jason Alexandra

Rowalt Alibudbud

Kasim Allel

Mutahar Ahmed Al-Qassimi

Stéphane Amadéo

Leopold Aminde

Jennifer Andersen

Craig Anderson

Melissa Andrew

Nadine Andrew

Yee Ang

Arman Arab

Ronen Arbel

Jun Arii

Giacomo Aringhieri

Mikko Arvas

Todd Anthony Astorino

Katie Attwell

Suzanne R Avis

Peter Baade

Louise Baandrup

Helena Backman

Wei Bai

Jodie Bailie

Daniel Bainbridge

Jocasta Ball

Lindley Barbee

Katherine Ann Barraclough

Jenny Barrett

Alfred Bartolucci

Lingkan Barua

Sudha Basnet

Buddha Basnyat

Hannah Bassett

Tarun Bastiampillai

Sina Bavari

Diana Beatriz Samson Bayani

Richard H Bayford

Torben Becker

Massimiliano Beghi

Leila Bell

Tal Ben-Ami

Wyatt P Bensken

Steven L Bernstein

Alvise Berti

Zulfiqar Bhutta

Peng Bi

Colin Binns

Giuseppe Biondi-Zoccai

S Jeff Birchall

Antony Black

Dirk Blom

Laura Boekel

Minyahil Tadesse Boltena

Kasper Bonnesen

Robert Booy

Rohan Borschmann

Louise Bourchier

Natalie Katrina Bradford

Nicholas Brink

Andrew Dwight Brown

Jennifer Browne

Antonio Brunetti

Lin Lin Bu

Jeffrey C Buchsbaum

Adrian Budhram

Dario Buioni

Shazna M Buksh

Danilo Buonsenso

Haewon Byeon

Siddappa Byrareddy

Dominique Cadilhac

Maria Stephanie Fay Samadan Cagayan

Jun Cai

Ziyi Cai

Giuseppe Caminiti

Jackie Campbell

Guillermo Arturo Cañadas-de la Fuente

Maria Laura Canale

Guangwen Cao

Hua Cao

Pianpian Cao

Wen-Ming Cao

Yifan Cao

Yun Cao

Claudia Cardoso

Neus Carrilero

Ben Carter

Maria Carvalho

Alessandro Cassini

Adriano Casulli

Jessica Catchpole

Goylette Chami

Joey Chan

Juliana Chan

Kei Hang K Chan

King-Pui Florence Chan

Kwok Ying Chan

Ngan Yin Chan

Polin Chan

Renee W Y Chan

Sherry Kit Wa Chan

Yean Yean Chan

Nomathemba Chandiwana

Yu-Mei Chang

Keith Chappell

Hadrien Charvat

Chuchih Chen

Jie Chen

Juliana Chen

Jun Chen

Mengni Chen

Peng Chen

Renjie Chen

Runsen Chen

Shanquan Chen

Shu Chen

Shujie Chen

Simiao Chen

Wanqing Chen

Wei-heng Chen

Xiaoxin Luke Chen

Yajun Chen

Yilung Chen

Yunbo Chen

Hao-Min Cheng

Jian Cheng

Lei Cheng

Xunjie Cheng

Lynne M Chepulis

Peter Cheung

Shengchu Chi

Chifa Chiang

Hannah Chisholm

Mohammod Jobayer Chisti

Cheng-Hsun Chiu

Sopio Chochua

Horace Choi

Eric Chow

Courtney Choy

Robert Christensen

Dinh-Toi Chu

Lingzhi Chu

Robert Clarke

Gary Clifford

Stephan Collishaw

Alex Cook

Andrew Copas

Keith Couper

Loes Crielaard

Julio Croda

Tony Cunningham

Megan Rose Curtis

Michael Curtis

Min Dai

Katie Dale

Kelsey Dancause

Thu Dang

Benjamin Daniels

Doan Dao

Enza D'Auria

Zoe E Davidson

Geertruida de Bock

Jeroen De Bont

Robert Jacobus de Knegt

Anthony De Soyza

Edward Dee

Artur Figueiredo Delgado

John Paul Caesar delos Trinos

Ender Demir

Guangtong Deng

Sahar Derakhshan

Michael Dewey

Qian Di

Danilo Di Bona

Julio Diaz

Joseph Dieleman

Changhai Ding

Ding Ding

Ning Ding

Xiaoqiang Ding

Genevieve Dingle

Nicola Disma

Padraig Dixon

Sabelo Nick Dlamini

Matthew Dodd

Guang-Hui Dong

Huiyu Dong

Jianzeng Dong

Yanhui Dong

Zhao Dong

Katina D'Onise

James Dowler

Houwei Du

Lingbin Du

Shufa Du

Xiaoyang Du

Yiqi Du

Jaime Duffield

Dorothea Dumuid

Matthew Dunn

Isabelle Durand-Zaleski

Katherine Duszynski

Alexandra Edelman

Harald Ehrhardt

Sarah Elshahat

Kamil Erguler

Claudia Estcourt

Paolo Eusebi

Michael Falster

Xiaoyan Fan

Hai Fang

Li-Qun Fang

Ya Fang

Yuan Fang

Zuyi Fang

Eric Faragher

M M A Faridi

Forough Farrokhyar

Majid Fasihi Harandi

Robin Fears

Daniel Feikin

Jamie Feldman

Ruimei Feng

Xing Lin Feng

Zhixin Feng

Zhongxiang Feng

Tanis Fenton

Israel Fernández-Cádenas

Pietro Ferrari

Natan Feter

Steffen Flessa

Joël Fokom Domgue

Chuan De Foo

Kathryn Foti

Louise Freijser

Hongqiao Fu

Lijun Fu

Jennifer Furin

Shannon Galyean

Mihir Gandhi

Fuqiang Gao

Pei Gao

Wei Gao

Yadong Gao

Marcial García

Jacopo Garlasco

Matteo Gastaldi

Mario Gaudino

Alan Frederick Geater

Meseret Gebre

Charlotte Genestet

C R Robert George

Francois Ghiringhelli

Rakhi Ghoshal

Maria Lorella Giannì

Stuart Gilmour

Maria Stella Giron

Lucy Goldsmith

Jessica Gong

Tomas Jose Gonzalez-Lopez

Felicity Goodyear-Smith

Heather Gordish-Dressman

Yoshikazu Goto

Jiangtao Gou

Igor Grabovac

Rebekah Graham

Giorgio Grani

Ruby Grant

Heather M Gray

Andreas Greinacher

Karen Grépin

Gregory Gromowski

Dongmei Gu

Xiaodong Guan

James Gulley

Erica Gunderson

Chao Guo

Snehil Gupta

Yashdeep Gupta

Akshay N Gupte

Deepti Gurdasani

Eunhee Ha

Luc Hagenaars

Omar Hahad

André Hajek

Samar Hajj

Simon Hales

Brian Hall

Jostein Hallén

Shinjiro Hamano

Chisato Hamashima

Sang Hoon Han

Shuping Han

Xiaolei Han

Daniel Hanley

Jim Hanly

Mainul Haque

Katie Harris

Emily Harrison

John Hart

David Häske

Alison Hayes

Ping He

Yulei He

Emma Heard

Harald Hegen

Arnfinn Helleve

Allison J Hempenstall

Dianna Hergott

Manuel Hetzel

Soawapak Hinjoy

Daniel Sai Yin Ho

Hung Chak Ho

Charlotte Hobbs

Janet Hoek

Nicolas Hoertel

Eric Hoffman

Markus Hoffmann

Timothy H Holtz

Kirsten Holven

Nusrat Homaira

Preben Homøe

Yoshikazu Honda-Okubo

Yoon-Ho Hong

Young-Rock Hong

Ria E Hopkins

Benjamin Horne

Elsie Horne

Jian Hou

Lei Hou

Zhiyuan Hou

Yingfen Hsia

Hui-Min Hsieh

Hao Hu

JianXiong Hu

Siqi Hu

Songhua Hu

Hairong Huang

He-Feng Huang

Huiyao Huang

Junjie Huang

Wei-Lieh Huang

Yi-Hsiang Huang

Yixiang Huang

Zhilian Huang

Zhiyong Huang

Chung-Lieh Hung

Ivan F N Hung

Muhammad Jami Husain

Giao Huynh

Zoë Hyde

Yoko Ibuka

Sindana Ilango

Sébastien Imbert

Lawrence Impey

Maki Inoue-Choi

Ana-Maria Iosif

Nicola Irwin

Chieko Ishiguro

Masanobu Ishii

Md Islam

Sheikh Mohammed Shariful Islam

Hidehiro Itonaga

Masao Iwagami

Caroline Jackson

Daniel Janitschke

Maneka Savithri Jayasinghe

Ravindran Jegasothy

Jianguang Ji

John Ji

Lu Ji

Jianping Jia

Peng Jia

Dong Jiang

Jingmei Jiang

Ke Jiang

Weixi Jiang

Yawen Jiang

Yixuan Jiang

Dong-Yan Jin

Heng Jin

Lei Jin

Jeremy Johnson

R Rima Jolivet

Kai Jonas

Katherine Jonas

Steven Julious

Zubair Kabir

Shigenori Kadowaki

Aimilia Kallitsounaki

Hidehiro Kaneko

Hyun Kang

Kaliaperumal Karthikeyan

Yasuhiro Kato

Atsushi Kawakami

Yoshihiro Kawaoka

Margaret Kay

Alex Kayongo

Negin Kazemian

Yang Ke

Karen Keddy

Roya Kelishadi

Christopher Kellner

Bridget Kelly

Sherrie Kelly

Mark Kelson

Zoe Kelson

Anna Kemp-Casey

Alex R Kemper

Syed Afroz Keramat

Winfried Kern

Thet Khaing

Mohamed Khalis

Safi Khan

Ali Khashan

Resham Khatri

Swee Kheng Khor

Stefan Kiechl

Beom Kyung Kim

Hyeon Chang Kim

Kwang-il Kim

Kyae Hyung Kim

Tae Hyun Kim

Miyako Kimura

Taishiro Kishimoto

Janet Kist

Duleeka Knipe

Janwillem Willem H Kocks

Bogda Koczwara

Mariko Kogo

Jill Kolesar

Xiangqing Kong

Marko Korhonen

Karel Kostev

Konstantinos Kostikas

Tomomi Kotani

Ebrahim Kouhsari

Sari Kovats

Thomas Kovesi

Akiko Kowada

Yuriko N Koyanagi

Evangelos Kritsotakis

Yi-Qun Kuang

Amit A Kulkarni

Takumi Kumai

Daisuke Kurai

Junya Kuroda

Naoto Kuroda

Jelena Kuvač Kraljević

Tsz Ho Kwan

Seunghyun Kwon

Shwe Sin Kyaw

Kefang Lai

Xiaozhen Lai

Sham Lal

Hugh Lam

Kyle Lam

Caroline Lambert

Julio Alejandro Lamprea-Montealegre

Manyu Lan

Peter Lange

Mark Larsen

Jerrald Lau

Lincoln Lau

Michael Law

Judith Lawrence

Flora Le

Chau Shoun Lee

Eng Sing Lee

Eun-Young Lee

Hae-Young Lee

Hyejin Lee

Ji-ho Lee

Jin Yong Lee

Jin Yong Lee

Jong-hyuk Lee

Sangjune Lee

Siwoo Lee

Victor Lee

Yew Jin Lee

Yong Yi Lee

Yunhwan Lee

Clara Lehmann

Xinyi Leng

Stuart Leske

Janni Leung

Grant Lewison

Ang Li

Chunsheng Li

Fei Li

Guanqiao Li

Huai-chen Li

Jie Li

Jing Li

Jinghua Li

Jingxin Li

Jiong Li

Lanjuan Li

Liming Li

Lin Li

Liwen Li

Na Li

Ni Li

Qian Li

Qun Li

Shizhu Li

Shoujun Li

Tiantian Li

Weimin Li

Wen-Qing Li

Xiaoping Li

Xin Li

Xue Li

Yigang Li

Yueyue Li

Zixiao Li

Yuanbo Liang

Rebecca Lillis

Lee Ling Lim

Chunqing Lin

Haishu Lin

Leesa Lin

Yung-Chieh Lin

Joni Lindbohm

Martin Lindström

Gregory Lip

Cheng-Cheng Liu

Hanruo Liu

Jue Liu

Kui Liu

Longding Liu

Mark C Liu

Min Liu

Qiyang Liu

Shiwei Liu

Yong Liu

Yuanyuan Liu

Zi-Yue Liu

Michael James Loftus

Denis Logunov

Thomas Longden

Catherine Lord

Hong Lu

Xin Lu

Yihan Lu

Yoel Lubell

Gregory Lucas

Alejandro Lucia

Tom Lung

Fengming Luo

Yanan Luo

Jun Lv

Shan Lv

Elizabeth A Lynch

Ronan Lyons

Mark Lythgoe

Jun Lyu

Davidh Hui Kang Ma

Hao Ma

Tianyi Ma

Yiming Ma

Ausenda MacHado

Alison Macpherson

Joerg Mahlich

Bernardo Mainou

Hei Wan Mak

Amyn Malik

Rainer Malik

François Maltais

Lenore Manderson

Olena Mandrik

Sarah Maness

Mohammad Ali Mansournia

Jonathan Mant

Adrian Marcato

J Marchi

Alessandro Marcon

Eloi Marijon

Virve Marionneau

Michael Marks

Juan Marquez-Romero

Charles Marshall

David Mataix-Cols

Jordi A Matias-Guiu

Tetsuya Matsubayashi

Fiona Matthews

M Pilar Matud

Etuini Ma'u

Dyah Samti Mayasari

Karen Mcbride-Henry

Malcolm I McDonald

Sally McManus

Claire Meek

Erik Meijer

Lana Meiqari

Yohannes Adama Melaku

Ritesh Menezes

Fiona Mensah

Benjamin Meyer

Melissa Mialon

Daniel Michaeli

Lars Michel

Jo Middleton

Philippa Middleton

Filippo Migliorini

Long Chiau Ming

Jonathan Minton

Alex Miras

Aniket Mishra

Tomoya Mita

Takeshi Miyazaki

Ann Beth Moller

Himel Mondal

Marco Montalti

Lindsey Monteith

José Manuel Montes

Sydney B Montesi

David Moore

Rosemary Morgan

Masafumi Moriyama

Chloe Morozoff

Nathan Morris

Christopher Morrison

Jedidiah I Morton

Filip Morys

James N Moy

Naaheed Mukadam

Thomas Munzel or Muenzel

Michio Murakami

Kamarul Imran Musa

Tomoya Myojin

Rong Na

Kari Christine Nadeau

Lucie Nadeau

Nico Nagelkerke

Nicolas Nagot

Atsushi Nakagomi

Eishin Nakamura

Yasuhiro Nakanishi

Yuemin Nan

Abdulqadir Nashwan

Cecilia Gretchen Navarro-Locsin

Takahiro Nemoto

John P Newall

Mark W Newton

Shu Wen Ng

Nhung Nghiem

Son Nghiem

Hoai Dung Thi Nguyen

Phuong Nguyen

Tan Nguyen

Eric Nilles

Kunihiro Nishimura

Yue Niu

Liz Noguera Zayas

Choong-Kyun Noh

Shuhei Nomura

John Norrie

Christiana Nöstlinger

Lena Novack

Rachel Nugent

José Nuño-Ledesma

Tommy Nyberg

Kerry-Ann O'Grady

Ayodele Ogunleye

Yukitaka Ohashi

Hiroyuki Ohbe

Motohiro Okada

Ryosuke Okamura

Tomonori Okamura

Frances Claire Onagan

Christine J ONeill

Eugenia Ong

Kanyin Ong

Win Han Oo

Tomiko Oskotsky

Yuichiro Otsuka

James C Overholser

Kei Owada

Peter Paal

Matthew Painschab

Pamela Palasanthiran

Jay Pan

Jin-Shui Pan

Xiao-chuan Pan

Yuesong Pan

Basu Dev Pandey

Amanda Panfil

Jing Pang

Daniel Park

Duk-Woo Park

Ali Parsa

Timothy Pawlik

Changmin Peng

Songxu Peng

Wen Peng

Pablo Perez-Guzman

Javier Perez-Saez

Vaios K Peritogiannis

Stephanie Perniciaro

Jennifer Perret

Benjamin Perry

Richard Perry

Hoang Phan

Paul D P Pharoah

Giorgina Barbara Piccoli

Edoardo Picetti

Yogan Pillay

Pascal Pineau

Zhiguang Ping

Carlos Piñones-Rivera

Elisabete Cristina Bastos Pinto

Mathias Pletz

Anju Poudel

Davoud Pourmarzi

Kumar Prabhash

Robin Prescott

Hong Qi

Haoqi Qian

Menbao Qian

Simon Xiwen Qin

Chao Qiu

Xinye Qiu

Zenghui Qiu

Frederick Raal

Angela Raffle

Delphine Rahib

Kazi Rahman

Yogambigai Rajamoorthy

Kollengode Ramanathan

Ramya Ramaswami

Mao-Sheng Ran

Jonas Ranstam

Otavio Ranzani

Huiying Rao

Krishna Rao

Aswin Ratheesh

Neda Razaz

Susan Rees

Steve Reid

Kyndaron Reinier

Ping Ren

Zhigang Ren

Ravi Retnakaran

Chin Kook Rhee

Alan S Rigby

Bart Rijnders

Jo Robinson

Benjamin Roche

Carlota Rodríguez García

Kris Rogers

Michael Roguski

Susanne Röhr

Fernando Rosell-Ortiz

Pierre-Marie Roy

Tobias Ruck

Nicolas Rüsch

Joanne Ryan

Sukhyun Ryu

Chan Hang Saing

Justin Salciccioli

Jon Salmanton-García

Carlos A Sánchez

Sven Sandin

Ailiana Santosa

Sondi Sararaks

Ponnusamy Saravanan

Celestino Sardu

Gautam Satheesh

Sebastian Schnaubelt

Philipp Schuetz

Michael Schwarzschild

Petar Seferović

Daniel I Sessler

Wai-Kay Seto

Arash Shaban-Nejad

Ayyoob Sharifi

Phil L Shearman

Mary C Sheehan

Chen Shen

Mingwang Shen

Fu-Dong Shi

Jing Shi

Ju-Fang Shi

Xuefei Shi

Yaojiang Shi

Kyoko Shimamoto

Kazuki Shimizu

Jaimie Shing

Masayoshi Shinjoh

Kohei Shitara

Sonia B Sia

Sameen Siddiqi

David Simmons

Sami Simons

Ravi Shankar Singh

J Mark Sloan

Alexander Smith

Johanna Snäll

Evan Snitkin

Hon-Cheong So

Natasha Sobers

Jung-kook Song

Ranran Song

Suhang Song

Yi Song

Sumire Sorano

Joan Soriano

Bogdan Marian Sorohan

Gemma Frances Spiers

Matthew Spittal

Shiranee Sriskandan

Naomi Ssenyonga

Baudouin Standaert

Kimberly A Stanford

Matthew Stargardter

Annalee Stearne

Mark Stokes

Caroline M Stretton

Zuzana Strizova

Elizabeth Sturgiss

Xiaoyou Su

Zhaohui Su

Elisabetta Suffredini

Daniel Sugrue

Giorgia Sulis

Pengming Sun

Qiang Sun

Shengzhi Sun

Yingxian Sun

Paweena Susantitaphong

Meinolf Suttorp

Aaron Sverdlov

Meisam Tabatabaei

Hirokazu Tachikawa

Angela Taft

Marco Tagliamento

George Taiaroa

Soichi Takeishi

Masato Takeuchi

Victor Talisa

Nicholas Talley

Mohammad Radwanur Talukder

Koku Sisay Tamirat

Celine Tan

Kelvin Bryan Tan

Atsushi Tanaka

Junko Tanaka

Mitsunobu Tanaka

Mototsugu Tanaka

Jie Tang

Shixing Tang

Weiming Tang

Wenxi Tang

Ourlad Alzeus Tantengco

Peter Taylor

Chloe Teasdale

Andrea Teng

Alvin Kuo Jing Teo

Phong Thai

Yih-Chung Tham

Caroline Thng

Chloe Thomas

Rhiannon Thompson

Simon James Thornley

Jim Thornton

Ariadna Tibau

Simon Timpka

Chuo Yew Ting

Kelvin To

Shweta Todkar

Shilu Tong

Yongsheng Tong

Thaddäus Tönnies

Daniel J Tozer

Takehiro Tozuka

Colm Travers

Adam Trickey

Vincenzo Trischitta

Nicole Ngai Yung Tsang

Apostolos Tsiachristas

Vivien Tsu

Kenichi Tsujita

Shinya Tsuzuki

Wen-Jun Tu

Claire Turner

Hugo Turner

Peter Turner

Greg Tyrrell

Kazuhiro Uda

Chi-Chi Udeagu

Thomas Unger

Akiyuki Uzawa

Günalp Uzun

Oğuz Uzun

Arschang Valipour

Alexandre Vallée

Bernadette Wilhelmina Antonia van der Linden

H Rogier van Doorn

Jennifer Ilo Van Nuil

Nicolien van Ravesteyn

Annelies van Rie

Mandira Varma-Basil

Priscila A Vasconcelos

Vidanka Vasilevski

Andrey Vasin

Byron Vaughn

Ravindra Veeranna

Natanasabapathy Velmurugan

Timo Vesikari

Viktor von Wyl

Alisha Wade

Kayo Waki

Susan Waller

John Walley

Xiaomin Wan

Yanmin Wan

Chongjian Wang

Debin Wang

Dongfang Wang

Honghong Wang

Jianming Wang

Jiguang Wang

Kailu Wang

Ling Wang

Peng Wang

Pengfei Wang

Qing Wang

Qun Wang

Ruoyu Wang

Shaobin Wang

Shi-Heng Wang

Shuhang Wang

Siwen Wang

Wenhua Wang

Xiang Wang

Xiao Wang

Xuemei Wang

Yang Wang

Ying Wang

Yixin Wang

Yongjun Wang

Youxin Wang

Yuan Yuan Wang

Yu-Chun Wang

Zhongfang Wang

Mary Njeri Wanjau

Iain Gordon Ward

Jeremy Warner

Nicholas Watts

Wei Wei

Xiaolin Wei

Yongyue Wei

Paul Weiss

Denggui Wen

Xue Wen

Xuerong Wen

Ronny Westerman

Dennis Wienand

Anna Wilkinson

Nick Wilson

Laura Winchester Winchester

S Tony Wolf

Eliza L Y Wong

Sabrina Wong

William W L Wong

Anna J Wood

Chao Wu

Chenkai Wu

Chi Shin Wu

Hongjiang Wu

Jiayuan Wu

Junyan Wu

Peng Wu

Rui Wu

Shaohong Wu

Ting Wu

Xinbao Wu

Zunyou Wu

Lei Xia

Ningshao Xia

Yu-Tao Xiang

Hong Xiao

Pei Xiao

Xiang Xiao

Yonghong Xiao

Li Xie

Yang Xie

Yue Xie

Zhengde Xie

Hualei Xin

Weijie Xing

Gelin Xu

Haiquan Xu

Jiancheng Xu

Jian-wei Xu

Jin-fu Xu

Liang Xu

Mingfang Xu

Pingyi Xu

Richard Huan Xu

Wanghong Xu

Weize Xu

Xiaolin Xu

Yu Xu

Ziyan Xu

Jason Yam

Chika Yamada

Hiroshi Yamaguchi

Mirian Yamaguchi

Yugo Yamashita

Zuoqin Yan

Bo-Yi Yang

Haomin Yang

Juan Yang

Jun Yang

Li Yang

Lianping Yang

Muh-Hwa Yang

Shigui Yang

Xilin Yang

Xueyan Yang

Yang Yang

Yin Yang

Yao Yao

Meiling Yap

Kazuo Yashima

Hussein Yassine

Pengpeng Ye

Shuang Ye

Jun-Jun Yeh

Eng-Kiong Yeoh

Anna Yeung

Wing Fai Yeung

Aiwen Yi

Vasoontara Sbirakos Yiengprugsawan

Jia Yin

Zundong Yin

Paul Yip

WanFen Yip

Changhoon Yoo

Eiji Yoshioka

Bin Yu

Bin Yu

Jin-Tai Yu

Quan Yuan

Jingli Yue

Ki Wook Yun

Ken Kin Lam Yung

Cameron J Zachreson

Zafar Zafari

Dorota Zarębska-Michaluk

Ruth Zaslansky

Jan Maciej Zaucha

Laurie Zawertailo

Yan Zeng

Yiqiang Zhan

Anao Zhang

Bo Zhang

Fengqing Zhang

Guwei Zhang

Hongxia Zhang

Jianduan Zhang

Jun Zhang

Kebiao Zhang

Longxian Zhang

Luxia Zhang

Min Zhang

Ming Zhang

Qingpeng Zhang

Shihui Zhang

Weisen Zhang

Yong Zhang

Zhonghan Zhang

Dong Zhao

Hehe Zhao

Yang Zhao

Yaxin Zhao

Guoqiao Zheng

Yang Zheng

Baoliang Zhong

Huaiyang Zhong

Huifang Zhou

Jianbo Zhou

Jian-Bo Zhou

Liang Zhou

Runhong Zhou

Zhen Zhou

Beibei Zhu

Bin Zhu

Chenjing Zhu

Chuanlong Zhu

Kangmin Zhu

Tao Zhu

Yifan Zhu

Yixiang Zhu

Zhen Zhu

Huachun Zou

Kun Zou

Zhiyong Zou

